# Challenges in Real-Time Prediction of Infectious Disease: A Case Study of Dengue in Thailand

**DOI:** 10.1371/journal.pntd.0004761

**Published:** 2016-06-15

**Authors:** Nicholas G. Reich, Stephen A. Lauer, Krzysztof Sakrejda, Sopon Iamsirithaworn, Soawapak Hinjoy, Paphanij Suangtho, Suthanun Suthachana, Hannah E. Clapham, Henrik Salje, Derek A. T. Cummings, Justin Lessler

**Affiliations:** 1 Department of Biostatistics and Epidemiology, School of Public Health and Health Sciences, University of Massachusetts—Amherst, Amherst, Massachusetts, United States of America; 2 Department of Disease Control, Ministry of Public Health, Bangkok, Thailand; 3 Bureau of Epidemiology, Department of Disease Control, Ministry of Public Health, Bangkok, Thailand; 4 Department of Epidemiology, Johns Hopkins Bloomberg School of Public Health, Baltimore, Maryland, United States of America; 5 Emerging Pathogens Institute, Department of Biology, University of Florida, Gainesville, Florida, United States of America; Santa Fe Institute, UNITED STATES

## Abstract

Epidemics of communicable diseases place a huge burden on public health infrastructures across the world. Producing accurate and actionable forecasts of infectious disease incidence at short and long time scales will improve public health response to outbreaks. However, scientists and public health officials face many obstacles in trying to create such real-time forecasts of infectious disease incidence. Dengue is a mosquito-borne virus that annually infects over 400 million people worldwide. We developed a real-time forecasting model for dengue hemorrhagic fever in the 77 provinces of Thailand. We created a practical computational infrastructure that generated multi-step predictions of dengue incidence in Thai provinces every two weeks throughout 2014. These predictions show mixed performance across provinces, out-performing seasonal baseline models in over half of provinces at a 2 month horizon. Additionally, to assess the degree to which delays in case reporting make long-range prediction a challenging task, we compared the performance of our real-time predictions with predictions made with fully reported data. This paper provides valuable lessons for the implementation of real-time predictions in the context of public health decision making.

## Author Summary

Predicting the course of infectious disease outbreaks in real-time is a challenging task. It requires knowledge of the particular disease system as well as a pipeline that can turn raw data from a public health surveillance system into calibrated predictions of disease incidence. Dengue is a mosquito-borne infectious disease that places an immense public health and economic burden upon countries around the world, especially in tropical areas. In 2014 our research team, a collaboration of the Ministry of Public Health of Thailand and academic researchers from the United States, implemented a system for generating real-time forecasts of dengue hemorrhagic fever based on the disease surveillance reports from Thailand. We compared predictions from several different statistical models, identifying locations and times where our predictions were accurate. We also quantified the extent to which delayed reporting of cases in real-time impacted our predictions. Broadly speaking, improving real-time predictions can enable more targeted, timely interventions and risk communication, both of which have a measurable impact on disease spread in epidemic and pandemic scenarios. It is vital that we continue to build knowledge about the best ways to make these forecasts and integrate them into public health decision making.

## Introduction

Producing accurate and actionable forecasts of infectious disease incidence at short and long time scales will improve public health response to outbreaks. Real-time forecasts of infectious disease outbreaks can facilitate targeted intervention and prevention strategies, such as increased healthcare staffing or vector control measures. However, we currently have a limited understanding of the best ways to integrate forecasts into real-time public health decision-making.

Dengue is a mosquito-borne infectious disease that places an immense public health and economic burden upon countries around the world, especially in tropical areas. A severe form of the disease, dengue hemorrhagic fever (DHF), may lead to debilitating pain, organ shock, and even death [1]. Currently over 2.5 billion individuals worldwide are at risk of infection with dengue, a mosquito-borne RNA virus [2]. Global incidence of dengue has increased significantly over the past few decades, with estimated annual global incidence of about 400 million infections each year [3].

Dengue is endemic in Thailand, which has 77 provinces including one large municipality (Bangkok). National annual incidence rates of reported dengue in Thailand range between 30 cases per 100,000 population and 224 cases per 100,000 population [4]. Some estimates suggest that between 50–80% of cases are inapparent and hence are difficult to detect clinically and often go unreported [5–7]. Annual outbreaks show dynamic temporal and spatial patterns, with great year-to-year and across-province variation, making public health planning and resource allocation an ongoing challenge [8, 9].

With the maturation of disease surveillance and reporting systems in recent years, real-time disease forecasting has become a realistic goal in some settings. Recognizing the importance of this emerging field, several governmental agencies have established disease prediction contests in recent years, with the goal of having contestants produce accurate forecasts: e.g. a 2013 CDC influenza prediction challenge [10], a 2014 DARPA chikungunya prediction challenge [11], and a 2015 National Science and Technology Council interagency Working Group dengue prediction challenge [12]. However, researchers and practitioners are still working to understand and establish a set of best practices for implementing real-time prediction algorithms in practice.

Creating predictions in real-time poses logistical, computational, and statistical challenges. Logistically, raw data must be made available in a standard format for processing into analysis datasets. Historical data is also needed to allow for training of the prediction model(s). To enable transparent evaluations, predictions should be formally registered and archived in a publicly available database. Computational infrastructure is needed to transform and/or merge raw data into the analysis dataset and to run the models themselves. Analytical challenges include appropriate model training, selection, and validation, considering adjustments for delayed or incomplete case reporting. Depending on the methods used, additional statistical work may be necessary to accurately report uncertainty in the reported predictions. Below, we describe our approaches to dealing with these challenges.

In this manuscript we present the results from the first year of forecasting DHF across the 77 provinces in Thailand. In 2014 our research team, a collaboration of the Ministry of Public Health of Thailand and researchers from multiple academic institutions, implemented a system for generating real-time forecasts of DHF based on current disease surveillance reports from Thailand. This paper illustrates several key components of a rigorous real time prediction framework, including:

- a reliable pipeline for data transfer, cleaning, and analysis, with a data storage architecture that can recreate datasets that were available at a particular time (Section 2),
- a statistical model of disease transmission used to generate real-time predictions of infectious disease incidence (Section 3),
- an appropriate and rigorous model validation framework, including aggregating evaluations across location, calendar time, and prediction horizon (Section 4), and
- an assessment of the impact of case reporting delays on the accuracy of predictions (Sections 3.3 and 4.2).

Valuable efforts have been made to create, validate, and operationalize real-time influenza predictions for the US [13], although these efforts have not faced the same challenges of systematic delays in reported data. The infrastructure that we present in this manuscript provides valuable lessons for other collaborative prediction efforts between public health agencies and academic partners.

## Methods

### Data overview

The data presented here come from the national surveillance system run by the Ministry of Public Health in Thailand. Monthly dengue hemorrhagic fever (DHF) case counts for each province are available from January 1968 through December 2005. Individual case reports (hereafter referred to as “line-list” data) were available for dengue fever (DF), DHF, and dengue shock syndrome from January 1, 1999 through December 31, 2014. The line-list data contains information on each case, including date of symptom onset, home address-code of the case (similar to a U.S. zip code), disease diagnosis code, and demographic information (sex, marital status, age, etc.). In years where we had overlapping sources for case data, the line-list data were used. A summary of province-level characteristics for all provinces in Thailand is provided in Table C in S1 Appendix. Since 1968, several provinces have split into multiple provinces. Details on how we accommodate these province separations are available in Table D in S1 Appendix. In one instance, the counts associated with a province (Bueng Kan) that split from another (Nong Khai) in 2011 have continued to be counted with the original province since we do not yet have enough data to predict for the new province.

Theoretical work demonstrates that by choosing the generation time as the discrete time interval for case reporting, the case reports may more easily be used to model the reproductive rate of the disease [14]. The generation time for dengue is two weeks, hence we aggregated the line-list data into biweekly intervals and interpolated the monthly counts into biweekly counts. (We used a definition of biweeks that followed a standardized definition based on calendar dates. See Table A in S1 Appendix). Interpolation was performed by fitting a monotonically increasing smooth spline to the cumulative case counts in each province, and then taking the differences between the estimated cumulative counts at each interval as the number of incident cases in a given interval.

We chose to use only DHF cases because: (1) DHF is more consistently reported across the 47 years of data collection, (2) DHF is less likely than DF to be misdiagnosed or to be differentially detected over time, and (3) from a public health perspective, DHF is a more relevant outcome, as it is a life-threatening condition and requires medical attention.

The analysis was conducted using the R language. [15] Data and code related to the analysis are available at https://github.com/reichlab/dengue-thailand-2014-forecasts.

### Ethics statement

The research aspects of this study were approved by the Johns Hopkins Bloomberg School of Public Health and University of Massachusetts Amherst institutional review boards. Patient data was analyzed anonymously.

### Real-time data management

**Fig 1:**
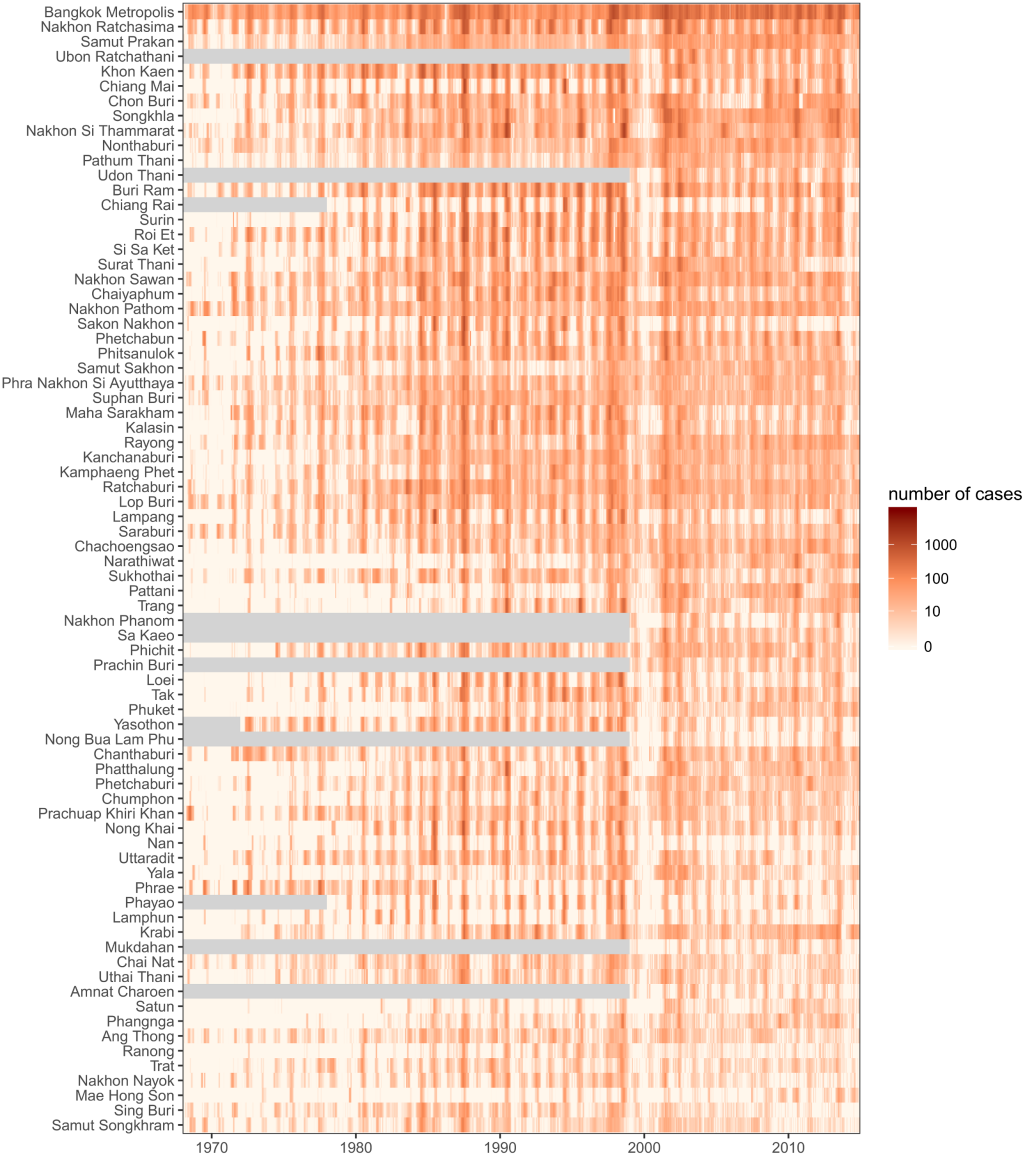
Raw dengue hemorrhagic fever case counts for 77 provinces of Thailand across 47 years (1968–2014). Provinces are ordered by by population (larger populations on the top). Gray regions indicate periods of time when a province was not in existence.

We established a secure data transfer process to transmit data from the Thai disease surveillance system to U.S. researchers. Throughout the 2014 calendar year, Thai public health officials transmitted data approximately every two weeks to a secure server based in Baltimore, Maryland (Table B in S1 Appendix). These data were then loaded into a PostgreSQL database containing all of the data, including monthly case counts and a table with all line-list data received to date. The final report containing a cleaned and finalized record of all cases for the 2014 season was delivered in April 2015. As of that time, this database held records of 2,503,631 unique cases of dengue in Thailand for the years 1968 through 2014, including records of 2,029,326 DHF cases (Fig 1).

When forecasting, we will only ever have the cases recorded prior to the time the predictions are made. So that we could compare the expected real-time performance of models as if they had been applied in real-time, all data were archived in the database with a time-stamp on arrival. This enabled researchers to “turn back the clock”, i.e. to query data that was available at a particular point in time. We refer to an “analysis date” as the date at which a multi-step forecast was made, using available data. Throughout this manuscript, we use the term “nowcast” to refer to predictions made for timepoints on or prior to the analysis date and “forecasts” to refer to predictions made for timepoints at or after the analysis date.

### Accounting for delays in case reporting

A key property of a surveillance system is the reporting delay, defined for our purposes as the duration of time between symptom onset and the case being available for analysis. During 2014 reporting delays for dengue ranged from 1 to 50 weeks. This was due to the process of reporting cases. Case reports typically follow a path of reporting from hospitals to district surveillance centers and then to provincial health offices before arriving at the national surveillance center. In all provinces, 50% of cases were reported within 5 weeks and 75% of cases were reported within 6 weeks. However, a small fraction of cases took quite a bit longer. To account for reporting delays, our models specified a reporting lag *l*, in biweeks. Data with onset dates within last *l* biweeks were considered to be not fully reported and left out from the analysis. We present results from the models with a lag of 6 biweeks (about 3 months), as these produced stable predictions across the entire country. More sophisticated adjustments for reporting delays are the subject of our team’s ongoing research.

### Timing of predictions

While the predictions presented in this manuscript were made retrospectively, in 2015 when complete data were available, they were constructed to mimic real-time predictions by using only the data available at each analysis date in 2014. During the 2014 calendar year, predictions from a similar model were generated in real-time and disseminated to the Thai Ministry of Public Health. We chose the set of analysis dates as the first day of each biweek for which data had been delivered in the previous biweek (Table B in S1 Appendix). For each analysis date in 2014, we used the candidate model to generate “real-time” province-level biweekly predictions for the subsequent 10 biweeks (5 months).

### Disease model: Features and estimation

#### Statistical model

We assumed the biweekly province-level reported cases follow a Poisson distribution, where the previous biweek’s reported cases serve as an offset term. Let the number of cases with onset occurring within time interval *t* in province *i* be represented as a random variable *Y*_*t*, *i*_, then

$$\begin{array}{ccc} Y_{t,i} & ∼ & Poisson(\lambda_{t,i}\cdot y_{t-1,i}) \end{array}$$

where the lag-1 term *y*_*t* − 1, *i*_ is used as an offset in this model. We adopt the convention of using lower-case *y*_*t*, *i*_ to indicate previously observed case counts that are treated as fixed inputs in our model. We explicitly model the rate *λ* as

$$\begin{array}{ccc} \log\lambda_{t,i} & = & f_{i}\left(b\left(t\right)\right)+g_{i}\left(t\right)+\sum_{j\in C}\sum_{k\in L}\alpha_{j,k}\log\frac{y_{t-k,j}+1}{y_{t-k-1,j}+1} \end{array}$$ (1)

where C is the set of *J* most-correlated provinces with province *i* and L is the set of lag times used in the model; *b*(*t*) is the biweek of time *t*; *f*_*i*_(*b*(*t*)) is assumed to be a province-specific cyclical cubic spline with period of one year (i.e. 26 biweeks); and *g*_*i*_(*t*) is a province-specific smooth spline to capture secular trends over time. Adding 1 to the numerator and denominator of the correlated province covariates ensures that the quantities are defined when no case counts are observed at a particular province-biweek. This method of adjusting for zero counts has been interpreted as an “immigration rate” added to each observation [16].

We note that the model can be expressed as

$$\lambda_{t,i}=E [\frac{Y_{t,i}}{y_{t-1,i}}|y_{t-1,i}]\approx R_{t,i}$$ (2)

which shows that *λ*_*t*, *i*_ can be interpreted as the expected reproductive rate at time *t* in location *i*, or *R*_*t*, *i*_ [14].

These models were fit using the Generalized Additive Model (GAM) framework (i.e. as generalized linear models with smooth splines estimated by penalized maximum likelihood) [17], using the mgcv package for R [15, 18]. Each province’s time-series was subset to remove any cases from the previous *l* biweeks. The remaining data were smoothed before fitting the model and making predictions.

Seasonal patterns were modeled using a penalized cubic regression spline, constrained to have a cycle of one year with continuous second derivatives at the endpoints. Secular trends were modeled using penalized cubic splines with 5 equally spaced knots over 47 years (roughly one knot per decade).

Information on epidemic progression elsewhere in the country was taken into account by including reported case counts at 1 lagged timepoint for the 3 most correlated provinces with province *i* in the data used to fit the model. Details of this model selection are provided in S1 Appendix.

We approximated the predictive distribution for all provinces using sequential stochastic simulations of the joint distribution of the case counts for each province. We created *M* independently evolving sequential chains of predictions by drawing, at each prediction time point, from the province-specific Poisson distribution with means given by eq (1). For example, if data through time t* was used to fit the models for all locations, then a single simulated prediction consisted of a simulated Markov chain of dependent observations for timepoints t* + 1, t* + 2, …, t* + *H*, across all provinces, where H was the largest horizon considered. To make a prediction for province *i* at time t* + *h* in the *m*^*th*^ chain, we draw

$${\hat{y}}_{t^{*}+h,i}^{m}∼Poisson\left({\hat{\lambda }}_{t^{*}+h,i}^{m}\cdot {\hat{y}}_{t^{*}+h-1,i}^{m}\right)$$

where ${\hat{\lambda }}_{t^{*}+h,i}^{m}$ is computed directly by plugging in the observed and predicted data prior to t* + *h* to the fitted model, and we use observed case data in the first step of prediction, i.e. ${\hat{y}}_{t^{*},i}^{m}=y_{t^{*},i}$ for all *m*. Due to the assumed interrelations between the provinces, we simulated counts for all provinces at a single timepoint before moving on to the next timepoint. For a given prediction horizon *h*, this process generates an empirical posterior predictive distribution for each province by evaluating the *M* different predictions for *y*_t*+*h*, *i*_. Prediction intervals are generated by taking quantiles (e.g., the 2.5% and 97.5%) of this distribution.

#### Metrics for evaluating predictions

We used several different metrics for evaluating our predicted case counts. We calculated Spearman correlation coefficients to measure the agreement between predicted and observed values. We also calculated the mean absolute error (MAE) by aggregating across analysis times within a given province. We computed the relative mean absolute error (relative MAE) comparing the predictions for a given province to predictions from a seasonal median baseline model. The seasonal baseline model for a given province is the median value of previously observed counts for the given biweek in that province over the past 10 years. The use of absolute error metrics over squared error metrics has been encouraged to enhance interpretability [19, 20]. Additionally, we calculated empirical 95% prediction interval coverage as the fraction of times the 95% prediction interval covered the true value.

#### Real-time vs. full-data predictions

**Fig 2:**
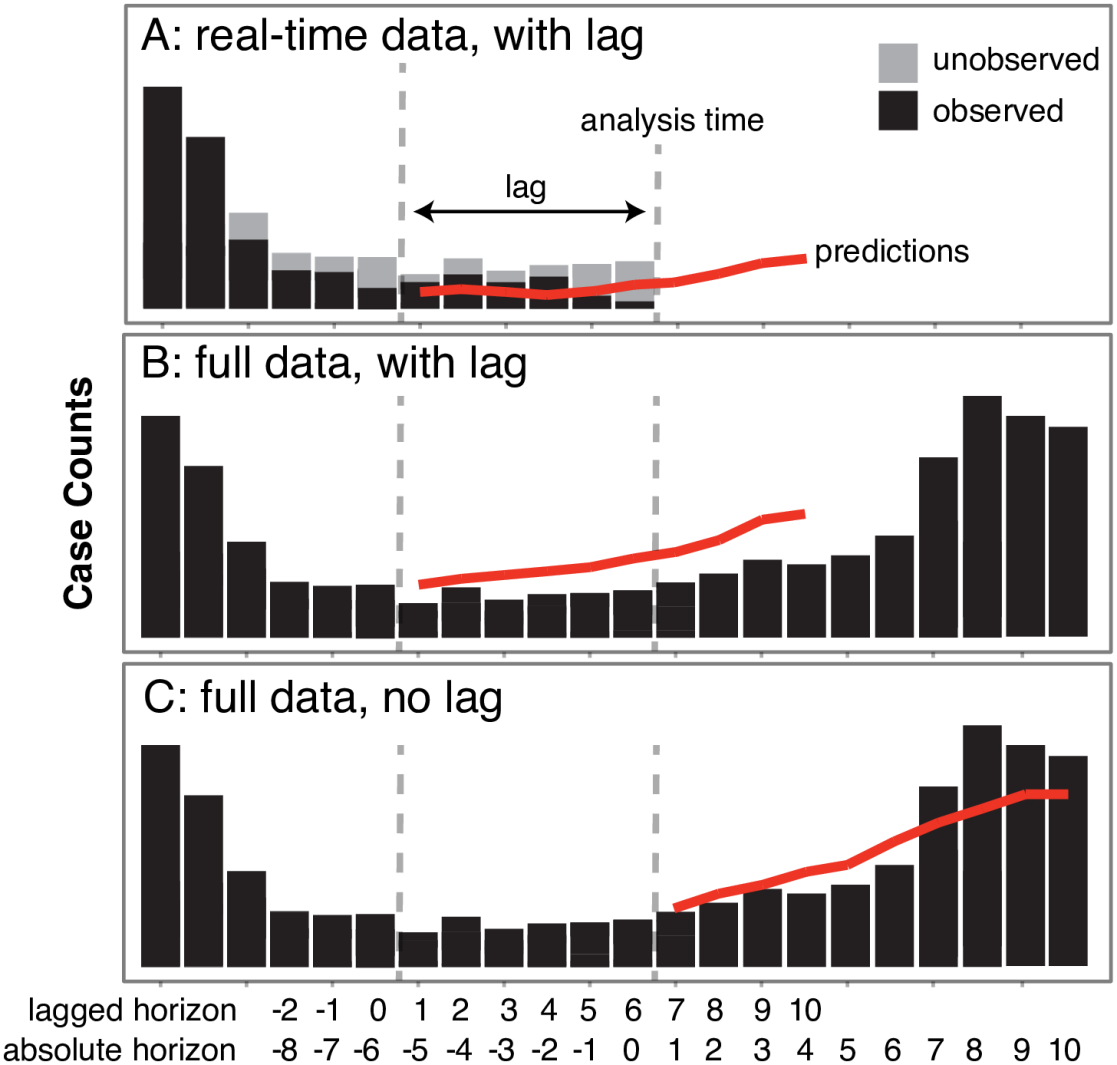
This figure illustrates three different methods used to create forecasts. Panel A shows predictions made using only data that was available at the analysis time, and ignoring the most recent six biweeks of reported cases. Panel B shows predictions that used fully observed data (including data that was not available at the analysis time) but still ignored cases from the six biweeks preceding the analysis time. Panel C shows predictions that could have been made at the analysis time if no reporting delays existed and all data that eventually was reported had been available in real-time.

We evaluated the performance of our real-time forecasts against predictions that could have been made had a full dataset been available at the analysis dates. To make this comparison, we ran a set of multi-step forecasts for 2014 at each analysis date using the complete data for 2014 that was finalized in late April 2015. We designed this experiment to focus on two comparisons. First, we aimed to compare real-time and full-data predictions where the multi-step predictions began at the same timepoint (Fig 2A vs. 2B). This analysis addressed the question of how much the real-time predictions were impacted by the delays in case reporting, even after beginning the predictions 3 months in the past. Second, we aimed to compare, by horizon, the real-time and full-data predictions where the origin of the multi-step full-data predictions was anchored at the analysis time but the origin of the real-time predictions was 6 biweeks earlier to account for delayed reporting of case data (Fig 2A vs. 2C). This analysis addressed the question of how much better or worse our model would have performed if full data were available without any delays.

## Results

### Summary of province-level forecasts

**Fig 3:**
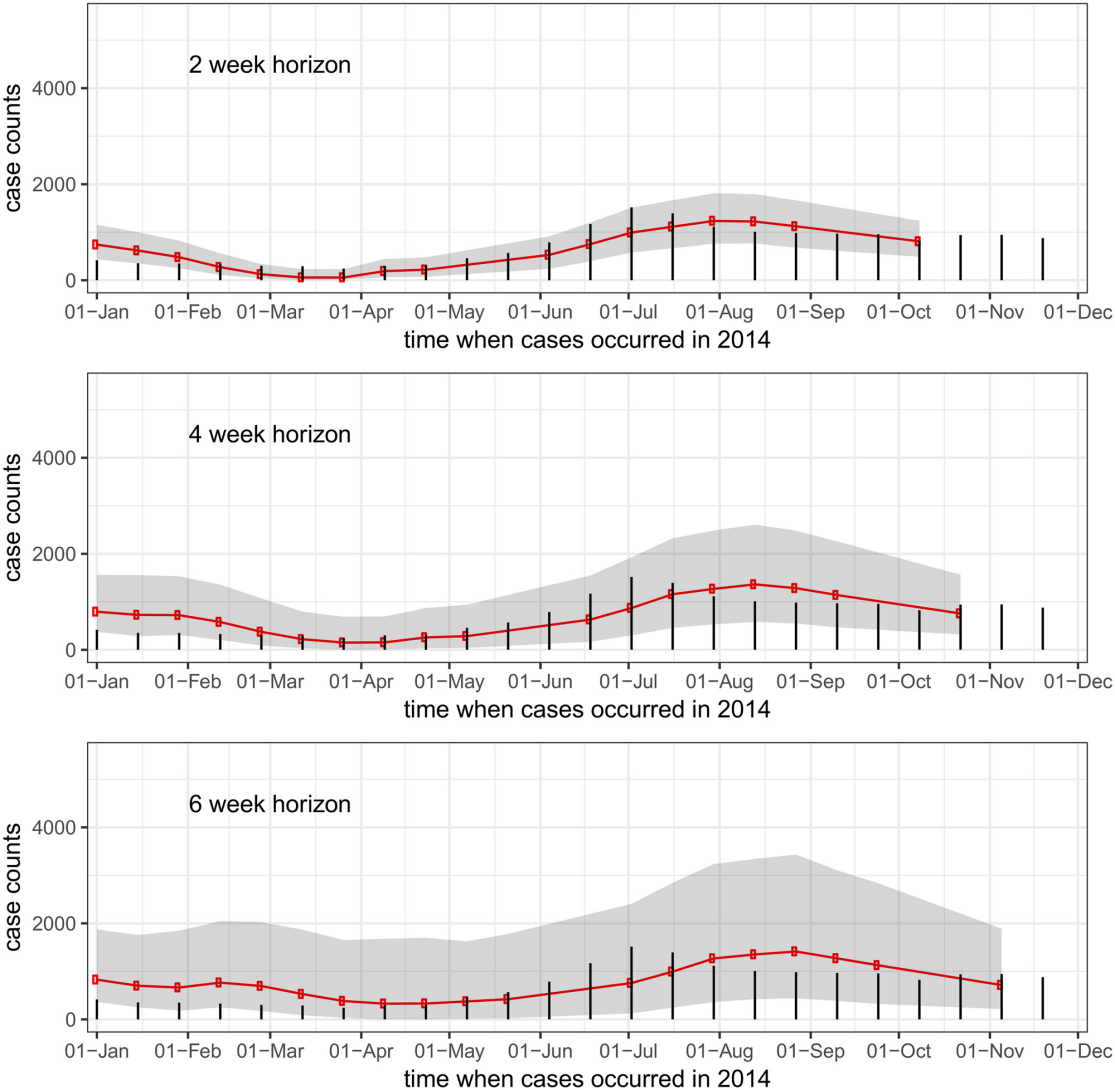
Country-wide real-time predictions for incident dengue hemorrhagic fever. Red lines show predicted case counts, black bars show cases reported by the end of the 2014 reporting period. The three figures show (top to bottom) one-, two-, and three-biweek ahead predictions. So, for example, every dot on the top graph is a one-biweek ahead real-time prediction made from all available data at the time of analysis.

In general, the model predictions showed good, if overconfident, performance at short horizons but less accuracy and high uncertainty at longer horizons. Across all provinces, the correlation between observed and predicted values was 0.89 at a horizon of 1 biweek (2 weeks) and 0.33 at a horizon of 10 biweeks, or approximately 5 months (see Table 1). Across all provinces, observed 95% prediction interval coverage was lower than expected at a horizon of 1 biweek (78%), showing that the models were overconfident in their short-term predictions. This prediction interval coverage increased to 97% at 4 and 5 biweeks (2-2.5 month) prediction horizon, and was near 95% for longer horizons. This indicates that our models often had an abundance of uncertainty at mid-term horizons. Fig 3 shows case counts and predictions aggregated across all provinces at horizons of 1, 2, and 3 biweeks (2, 4, and 6 weeks).

**Table 1:** Summary of real-time prediction accuracy, by prediction horizon. These results are aggregated across all provinces. The *R*^2^ and 95% PI coverage columns present the overall correlation coefficient and prediction interval coverage. The relative MAE columns show five quantiles of the distribution of province-level relative MAEs comparing the real-time model at the given horizon to a seasonal baseline model at the given horizon: *Q*_5_ (the 5th percentile), *Q*_25_ (25th percentile), *Q*_50_ (median), *Q*_75_ (75th percentile), and *Q*_95_ (the 95th percentile). The relative MAEs were calculated as the MAE from the real-time predictions divided by the MAE from the seasonal average predictions, therefore values larger than 1 indicate that the real-time models showed more absolute error on average than the seasonal models.

			relative MAE (real-time vs. seasonal baseline)				
horizon (h)	*R* ^2^	95% PI coverage	*Q*_5_	*Q*_25_	*Q*_50_ (median)	*Q*_75_	*Q*_95_
1	0.89	0.78	0.13	0.25	0.38	0.75	1.26
2	0.83	0.92	0.19	0.30	0.47	0.84	1.68
3	0.73	0.95	0.27	0.38	0.65	0.93	1.79
4	0.59	0.97	0.33	0.48	0.78	1.15	1.91
5	0.48	0.97	0.37	0.57	0.98	1.51	2.14
6	0.41	0.97	0.44	0.73	1.15	1.82	2.69
7	0.36	0.97	0.53	0.90	1.32	2.09	3.24
8	0.34	0.95	0.55	0.96	1.55	2.29	4.07
9	0.33	0.93	0.66	0.97	1.77	2.78	4.88
10	0.33	0.93	0.70	1.07	1.95	3.08	5.45

**Fig 4:**
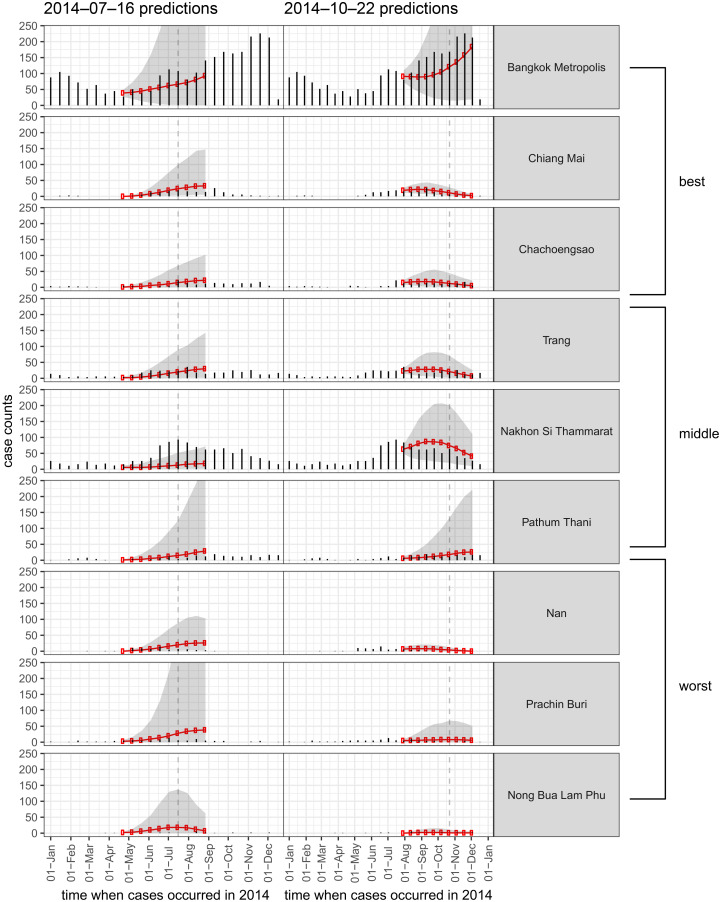
Ten-step forward predictions made with available data at two time-points in 2014 (each time indicated by a vertical dashed line). Results for nine provinces are shown, representing (from top to bottom) the best three provinces, the middle three, and the worst three performing provinces in terms of relative mean absolute error when compared to a seasonal baseline model.

Fig 4 shows examples of multi-step predictions from two analysis dates in 2014. We show the results from nine distinct provinces, representing the best three provinces, the middle three provinces, and the worst three provinces in terms of relative MAE when compared to a seasonal baseline model. The increasing uncertainty is visible in many cases, even when the point-predictions remain close to the true values. The explosive forecasts tended to occur more frequently in the early- and mid-season, when the historical seasonal trend rises and when the observed case counts tend to be increasing from one biweek to the next.

**Fig 5:**
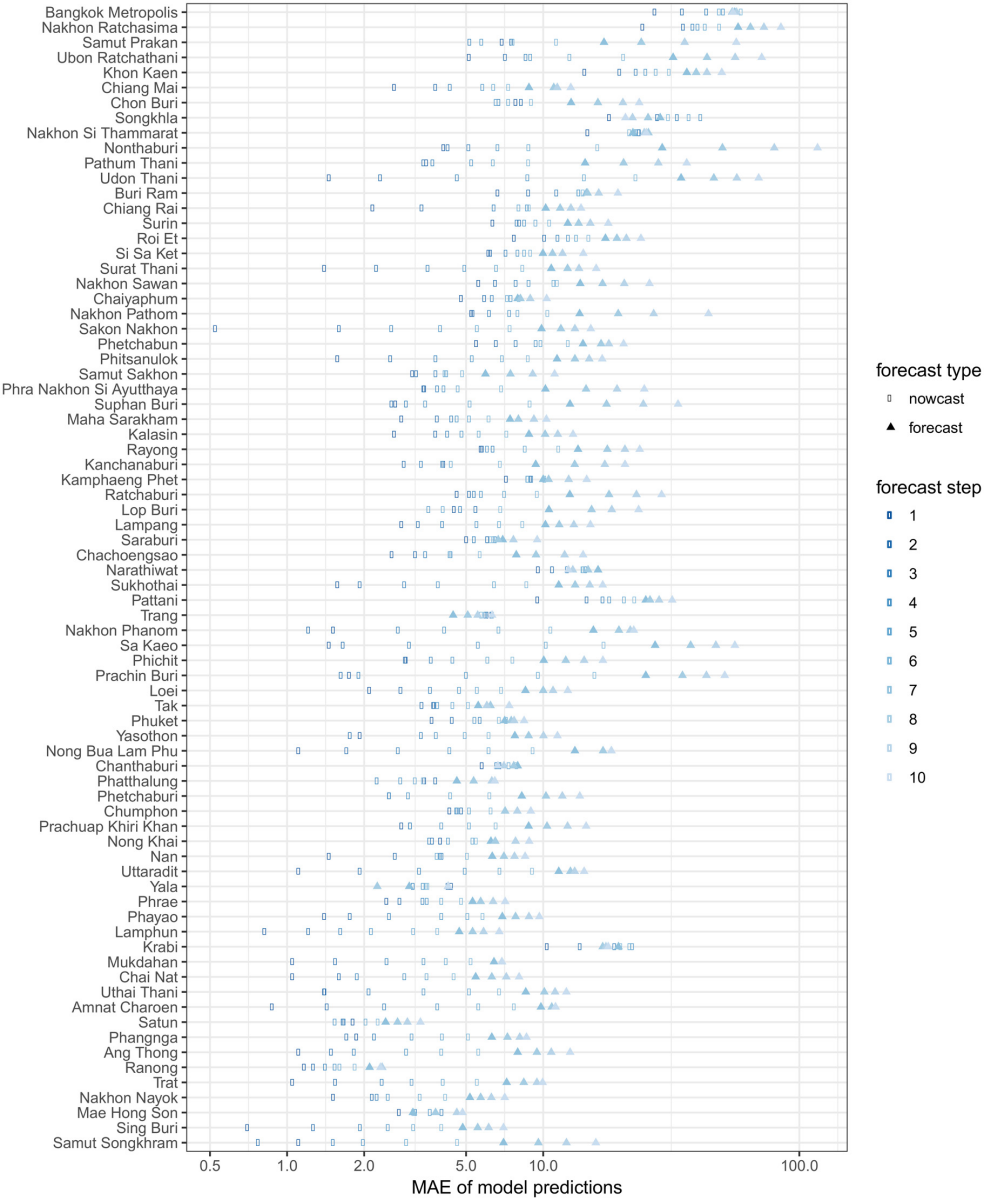
Mean absolute error (MAE) of our prediction model by province and step forward (in biweeks). Provinces are sorted by population, with the most populous at the top of the figure.

**Fig 6:**
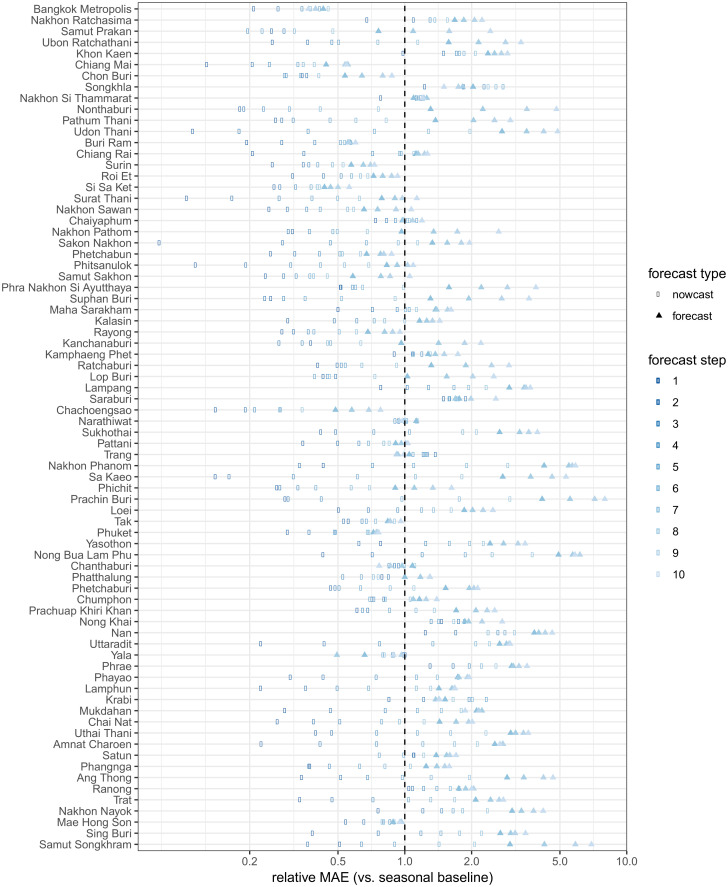
Relative mean absolute error (MAE) comparing our prediction model vs. a model that predicts a seasonal median, by province and step forward (in biweeks). Results to the left of the dotted line signify more accurate predictions from our models when compared to the seasonal model, and results to the right indicate less accurate predictions.

There was substantial variation in predictive performance across provinces. Mean absolute error (MAE) for predictions tended to be larger in provinces with higher populations (Fig 5), and also tended to increase with the forecast horizon. The observed MAE was less than 10 cases in over 90% of provinces at one time step and in over 50% of provinces at up to 6 time steps. Fig 6 shows the relative MAE of model predictions compared to a seasonal baseline model at prediction horizons of 1 through 10 biweeks (2 weeks through 5 months). We note that predictions during the first three months are nowcasts, as the most recent 6 biweeks of data are ignored in the fitting process and predictions are made starting from the point at which full data was assumed.

To compare predictive performance of our model between provinces, we used the relative MAE with a simple seasonal model as a baseline. Table 1 summarizes relative MAEs by prediction horizon. Relative to seasonal baseline prediction models, a majority of provinces made better predictions on average than the seasonal model at 1, 2, 3 and 4 biweek (2, 4, 6 and 8 week) prediction horizons (i.e. up to 2 months from the starting point of the predictions). Up to about 5 months from the origin of the multi-step predictions (and two months from the analysis time), over 15% of province-specific models made predictions that were on average better than the seasonal baseline model. Some province-specific models showed substantially worse predictions when compared to a seasonal baseline at these longer prediction horizons. No single province feature (e.g. total average cases, strength of seasonal trends, population size, season-to-season variation) was able to explain the substantial variations in performance, highlighting the challenges of creating a unified modeling framework for a set of varied locations (see S1 Appendix).

### Comparing real-time to full-data predictions

We compared real-time and full-data predictions that began at the same timepoint (Fig 2A vs. 2B). This analysis can help answer the question of how much the real-time predictions that removed the most recent 3 months of data were impacted by the delays in case reporting. As shown in Table 2, these analyses demonstrated that once we went back 3 months to begin the nowcasting, more than 50% of the provinces had more accurate real-time forecasts than full-data forecasts at all prediction horizons up to 1.5 months. This suggests that inaccuracies in the real-time predictions once those recent 3 months are discarded are driven less by the reporting delays than they are by model misspecification and other background noise in the data.

**Table 2:** Comparison of province-level prediction accuracy between full-data and real-time predictions, by prediction horizon. These calculations assume that both the full-data and real-time multi-step predictions began at the same time. The table shows the 5th percentile (*Q*_5_), 25th percentile (*Q*_25_), median (*Q*_50_), 75th percentile (*Q*_75_), and 95th percentile (*Q*_95_) value of the relative MAE from each province at the given horizon. The relative MAEs were calculated as the MAE from the real-time predictions divided by the MAE from the full-data predictions, i.e. values larger than 1 indicate that the real-time models showed more absolute error on average than the full-data models.

horizon	Relative MAE (real-time vs. baseline scores)				
	*Q*_5_	*Q*_25_	*Q*_50_ (median)	*Q*_75_	*Q*_95_
1	0.78	0.90	1.00	1.06	1.20
2	0.74	0.87	0.95	1.02	1.17
3	0.77	0.90	0.97	1.03	1.11
4	0.82	0.95	1.02	1.08	1.19
5	0.83	0.96	1.04	1.13	1.29
6	0.87	1.02	1.08	1.21	1.45
7	0.88	1.01	1.13	1.28	1.71
8	0.88	1.00	1.15	1.35	1.97
9	0.90	1.01	1.16	1.38	2.22
10	0.88	1.01	1.19	1.42	2.45

A second analysis compared real-time predictions with a horizon of 7 biweeks with full-data predictions at 1 biweek (Fig 2A vs. 2C). This analysis can tell us how much better or worse our model would have done if we did not need to adjust for delays in case reporting by dropping the past 3 months, i.e. if all of our data were available at the time of analysis. We refer to this realignment of horizons as the absolute horizon, to suggest that a real-time prediction that removes 6 biweeks of data and then projects 7 steps forward (Fig 2A) is predicting the same timestep as a full-data prediction that does not remove any data and just projects 1 biweek forward (Fig 2C). Results from this analysis are shown in Table 3 for absolute horizons of 1 through 4 biweeks. Overall, 74 of the 76 provinces (97%) showed better average performance in the full-data forecasts at 1 step ahead than the real-time forecasts at 7 steps ahead (i.e. had a relative MAE of greater than 1). In over 90% of the provinces at each absolute horizon the full-data forecasts were on average closer to the true value than the real-time forecasts. However across all the absolute horizons, for between 2 and 7 provinces the full-data predictions had more error than the real-time predictions. Full-data predictions under-performed real-time predictions in a small number of provinces, reflecting the challenges of making predictions in such a noisy system. A sample of predictions by province and analysis date are provided in S1 Appendix to illustrate this challenge.

**Table 3:** Comparison of province-level prediction accuracy between full-data and real-time predictions, by prediction horizon. These results were computed comparing predictions as if the full data was available at the analysis time with the real-time predictions that build in a 6-biweek (approximately 3 month) buffer to account for delayed case data. The table shows the 5th percentile (*Q*_5_), 25th percentile (*Q*_25_), median (*Q*_50_), 75th percentile (*Q*_75_), and 95th percentile (*Q*_95_) value of the relative MAE from each province at the given horizon. The relative MAEs were calculated as the MAE from the real-time predictions divided by the MAE from the full-data predictions, i.e. values larger than 1 indicate that the real-time models showed more absolute error on average than the full-data models.

Absolute horizon	Relative MAE (real-time vs. baseline scores)				
	*Q*_5_	*Q*_25_	*Q*_50_ (median)	*Q*_75_	*Q*_95_
1	1.37	1.77	2.81	5.55	11.26
2	1.01	1.61	2.89	4.88	12.23
3	0.98	1.68	2.94	4.25	9.55
4	0.92	1.83	2.86	4.14	8.74

## Discussion

We present the prediction results from our real-time prediction infrastructure established for dengue hemorrhagic fever in Thailand. This infrastructure addresses several key practical features of real-time predictions, including real-time data management, the impact of reporting delays, and incorporating a disease transmission model that takes into account spatial and temporal trends.

The infectious disease prediction literature has a rich and varied selection of prediction algorithms but has not historically focused on the challenges of generating predictions in real-time. Continued development and refinement of such prediction pipelines, such as that presented here, will enable existing prediction methods to reach their full potential in making an impact on public health decision-making and planning.

The infrastructure that we have developed for integrating real-time data into predictions for the Thai Ministry of Public Health (MOPH) is the result of a long-standing governmental/academic partnership between the MOPH and U.S.-based researchers. This collaboration has enabled the creation of a single, unified authoritative source of almost all governmental dengue surveillance ever collected in Thailand, dating back nearly 50 years [4]. Additionally, by enabling the transmitting of surveillance data in near real-time (every two weeks from October 2013 and continuing through the writing of this manuscript in 2016), this effort has created a valuable dataset that has catalogued the reporting delays in a live surveillance system. The predictions described in this manuscript were made available to the MOPH typically within two weeks of the data being delivered to the U.S. research team via a PDF report and a private, interactive web application. The MOPH has disseminated these results to provincial, regional, and national decision-makers for use in planning for and monitoring outbreaks. Moving forward, to maximize the use of these predictions, the forecasts will be presented at the monthly high-level meetings of MOPH authorities. Decision makers at the province or health region level will use these forecasts to inform decisions about launching new interventions. Designing studies to evaluate different methods of incorporating these forecasts into real-time decision-making is an area of ongoing research for our team.

Formal data archiving protocols should be followed when making real-time predictions. Real-time predictions should be (1) generated prior to having the final data available and (2) formally registered or time-stamped in an independent data repository. Taking these steps ensures that no bias (intentional or not) enters the scientific process of evaluating the predictions.

While we are actively developing and validating other prediction models for this data, we chose to report the results from the prediction model that we used during 2014 to provide draft predictions to Thai public health officials. We intentionally did not perform extensive *post hoc* model validation or evaluation to minimize the risk of overfitting our model to this particular dataset.

Our 2014 real-time predictions varied substantially by province in quality and public health utility. In over half of the Thai provinces, our model out-performed a seasonal baseline model predicting two months in advance. As the horizon moves forward, the seasonal baseline model makes better predictions in more provinces: at a 5 month horizon, just over 15% of provinces are predicted better by our model than the seasonal model.

Our ability to make effective predictions into the future in a majority of provinces is made difficult by delayed case reporting. Our analyses show that if there were no reporting delays, our model would make substantially more accurate predictions in nearly all of the Thai provinces (Table 3). In ongoing work, we are focusing efforts on building models that can create accurate “now-casts” of data, using other more readily available data to increase the accuracy of forecasts, an approach that has been implemented by other forecasting efforts [21].

While we have conducted extensive evaluation of the performance of our real-time predictions in 2014, this may not represent the performance of the model in other years. There is substantial year-to-year variation in annual province-level incidence in Thailand. The annual total number of cases observed in 2014 was in the lower half of previously observed annual incidence in 62 of 76 provinces. A complete characterization of our real-time model’s predictive performance will require evaluation across multiple years of data that is arriving in real-time, or with historical complete data with synthetically created reporting delays.

The simplicity of the statistical prediction models that we present in this manuscript are both a strength and a weakness. This type of phenomenological time-series model tends to show good predictive performance in the short term but have known deficiencies when making long-term predictions. Additionally, when forecasting forward from auto-regressive models, this can lead to instabilities and explosive forecasts, as was observed in the predictions from some of the provinces. Also contributing to the instability of our models in a prediction context are that we do not incorporate uncertainty in and use a smoothed value of the *y*_*i*, *t* − 1_ offset term.

The model that we present here has been shown to perform well in contexts where there are no reporting delays (results not shown). The auto-regressive model used in this work is based on a standard statistical auto-regressive integrated moving average (ARIMA) models. In fact, the reformulation of the ARIMA model in a disease transmission model context—making explicit the connection between modeling auto-regressive counts and the reproductive number, as shown in eq 2—is an important link between commonly used models in different fields. Model improvements under consideration include veryifying the utility of spatial features for all provinces, adding spatially smooth seasonal effects, choosing the correlated provinces serially through partial correlations, and incorporating overdispersion of case counts.

The past decade of biomedical research has borne witness to rapid growth in digital surveillance data. A pressing challenge for the professional and academic epidemiological and biostatistical communities is to learn how to turn this deluge of data into evidence that informs decision making about improving health and preventing illness at the individual and population levels. Improving real-time forecasts of infectious disease outbreaks is an important technical achievement, however, continued research and collaboration in this area is needed to develop a better understanding of how to communicate these results to public health decision makers and integrate infectious disease predictions into public health practice. The collaborative effort described by this manuscript provides a template for generating real-time predictions in practice and describes specific results from this effort to integrate modern tools of data science with public health decision making.

## Supporting Information

S1 Appendix Methodological details, supplemental figures and results. (PDF)
