# Correction: Challenges in Real-Time Prediction of Infectious Disease: A Case Study of Dengue in Thailand

**DOI:** 10.1371/journal.pntd.0010883

**Published:** 2022-10-26

**Authors:** Nicholas G. Reich, Stephen A. Lauer, Krzysztof Sakrejda, Sopon Iamsirithaworn, Soawapak Hinjoy, Paphanij Suangtho, Suthanun Suthachana, Hannah E. Clapham, Henrik Salje, Derek A. T. Cummings, Justin Lessler

## Notice of Republication

This article was republished on November 15, 2021, to correct an unintentional coding error that led to pervasive minor inaccuracies in the manuscript. Specifically, in processing and merging multiple real-time data files from the Thai Ministry of Public Health, duplicate case records of dengue were created when a single record should have been in place. This led to over-counting of cases in many periods of time, an error that impacted forecast models and evaluation of the forecasts themselves. Please download this article again to view the correct version.
