# Supplementary material for: Challenges in Real-Time Prediction of Infectious Disease: A Case Study of Dengue in Thailand
Source: PLoS Negl Trop Dis. 2016 Jun 15;10(6):e0004761. doi: 10.1371/journal.pntd.0004761 (PMC4909288; doi:10.1371/journal.pntd.0004761)
Supplement: S1 Appendix — (PDF) [file pntd.0004761.s001.pdf]

Supplemental Materials for:
Challenges in real-time prediction of infectious disease: a case study of dengue in Thailand

Nicholas G. Reich, Stephen Lauer, Krzysztof Sakrejda,
Sopon Iamsirithaworn, Soawapak Hinjoy, Paphanij Suangtho, Suthanun Suthachana,
Hannah Clapham, Henrik Salje, Derek A T Cummings, Justin Lessler

May 24, 2016

Contents

1 Statistical model details **1**
- 1.1 Definition of biweeks and analysis times 1
- 1.2 Province data management 3
- 1.3 Model selection 3
- 1.4 Methods for generating predictions 4
- 1.5 Comparisons of real-time and full-data predictions 4
- 1.6 Considerations in making real-time, multi-step predictions 4

2 Province-level factors that influenced predictive performance **6**

1 Statistical model details

1.1 Definition of biweeks and analysis times

For a given year, every date is mapped to a particular biweek in that year. We define the first biweek of every year as beginning on January 1st, at 00h00m00s and the last as ending on December 31st, 11h59m59s. Every year is defined to contain exactly 26 biweeks. To make predictions on the biweekly scale, daily case counts are aggregated into their respective biweek. Counts for biweeks that have 15 days are standardized by multiplying the count by $\frac{14}{15}$ and rounding to the nearest integer. The explicit Julian calendar day to biweek mapping is given in Table A.

A generic biweek b_k is defined as an interval $[t_k, t_{k+1})$ where t_k is the time where the biweeks begins (e.g. Jan 1, 00h00m00s) and t_{k+1} is the start of the next biweek. Every dataset is divided up into N bi-weeks (b_1 through b_N), each of either 14 or 15 days (see Table A).

Table A: Map of Julian days to biweeks used in data aggregation. Columns show the date a biweek starts and the duration for non-leap ("reg") and leap years.

biweek	reg_yr_datestart	reg_yr_dur	leap_yr_datestart	leap_yr_dur
1	Jan 01	14	Jan 01	14
2	Jan 15	14	Jan 15	14
3	Jan 29	14	Jan 29	14
4	Feb 12	14	Feb 12	14
5	Feb 26	14	Feb 26	15
6	Mar 12	14	Mar 12	14
7	Mar 26	14	Mar 26	14
8	Apr 09	14	Apr 09	14
9	Apr 23	14	Apr 23	14
10	May 07	14	May 07	14
11	May 21	14	May 21	14
12	Jun 04	14	Jun 04	14
13	Jun 18	14	Jun 18	14
14	Jul 02	14	Jul 02	14
15	Jul 16	14	Jul 16	14
16	Jul 30	14	Jul 30	14
17	Aug 13	14	Aug 13	14
18	Aug 27	14	Aug 27	14
19	Sep 10	14	Sep 10	14
20	Sep 24	14	Sep 24	14
21	Oct 08	14	Oct 08	14
22	Oct 22	14	Oct 22	14
23	Nov 05	14	Nov 05	14
24	Nov 19	14	Nov 19	14
25	Dec 03	14	Dec 03	14
26	Dec 17	15	Dec 17	15

Every forecast made specifies the following dates: a "to-date" (t_{to}), a "delivery-date" (t_{del}), and an "analysis-date" (t_{an}). The to-date specifies that the current forecast will only use cases whose symptom onset date is equal to or less than t_{to}. The delivery-date specifies that the current forecast will only use cases that were delivered on or before t_{del}. The analysis-date specifies when a given forecast was run.

To account for case reporting delays, our models specify a reporting lag l, in biweeks, which represents the number of biweeks back into the past for which data will be considered partially reported. In the forecasting models presented in this paper, these data are ignored. For example, if we received a data delivery in the biweek $b_k = [t_k, t_{k+1})$, then the forecast will assume that data for the past l whole biweeks are systematically underreported and that biweek b_{k-l-1} and all prior biweeks are complete. This process is documented in Figure A.

We chose the set of analysis dates as the first day of each biweek for which data had been delivered in the previous biweek (Table B).

Figure A: An example forecast timeline showing which cases are included relative to the delivery-dates and to-dates. In this figure, $l = 3$.

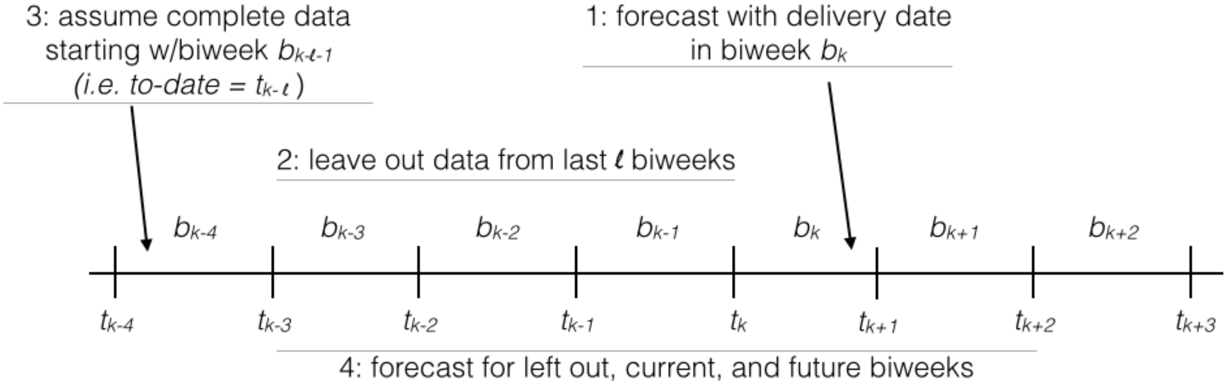

1.2 Province data management

Summary data on all provinces are provided in Table C.

Since 1968, five provinces were split into multiple provinces, from Yasothon breaking off from Ubon Ratchthani in 1972 to the foundation of Bueng Kan from Nong Khai in 2011 (see Table D for full list of split provinces). New provinces are labeled as "children" of the "parent" province from which they were formed. We used all available data for each child province - with the exception of Bueng Kan - though several of these did not start reporting dengue data for years after formation. For the parent provinces - with the exception of Nong Khai - we discarded all data before the first data year of their last child province. Since Bueng Kan is such a new province, we grouped all of its counts together with that of Nong Khai to keep one province rather than remove the provinces completely.

Since we observed that biweek 26 often appeared to have systematic underreporting even when all cases had been reported, we linearly interpolated the counts for the most recent biweek 26 prior to fitting any prediction model.

1.3 Model selection

Information on epidemic progression elsewhere in the country was taken into account by including reported case counts at various lags and for provinces that showed high levels of correlation with province i in the data used to fit the model. Each province considered itself as a possible province to choose but was not forced to include itself if other provinces showed higher correlation at the specified lag. We chose the number of top correlated provinces and lagged timepoints based on the combination that minimized country-wide leave-one-year-out cross-validation error between 2000 and 2009. We considered all possible combinations across a grid of 1 to 15 top correlated provinces and the following combination of lag times {(1), (1,2), (1, 2, 3), (1, 2, 3, 4), (1, 2, 3, 4, 13), (1, 2, 3, 4, 13, 26) }, where, for example, (1, 2, 3) refers to a model that included observations from top correlated provinces at lags of 1, 2, and 3 biweeks. Using the metric of relative mean absolute error with a reference model that predicted the last observed count, this process resulted in choosing 3 provinces at a 1 biweek lag

(a complete assessment of performance on fully observed data is in preparation). As shown in equation (1) in the main manuscript, these data enter the model as ratios. For example, the covariate for the lag-k biweek of province j for predicting a count at time t would be $\log \frac{y_{t-k,j}+1}{y_{t-k-1,j}+1}$.

1.4 Methods for generating predictions

To generate multi-step predictions of future unobserved timepoints, we created stochastic realizations of possible trajectories for each province. Specifically, our goal was to estimate the joint distribution $f(\mathbf{Y_{t^*+h}}|\mathcal{Y}_{\mathbf{t^*}})$ where t^* is the last time for which data was assumed to be fully observed, h is the target prediction horizon in biweeks, $\mathbf{Y_{t^*}}$ is a random vector of all province-specific counts at time t^*, and $\mathcal{Y}_{t^*}$ is the set of all observed $y_{t,i}$ where $t \leq t^*$ and for all i.

We approximated the predictive distribution for all provinces using sequential stochastic simulations of the joint distribution of the case counts for each province. We created M independently evolving sequential chains of predictions by drawing, at each prediction time point, from the province-specific Poisson distribution with means given by equation (1) in the main manuscript. For example, if data through time t^* was used to fit the models for all locations, then a single simulated prediction consisted of a simulated Markov chain of dependent observations for timepoints t^*+1, t^*+2, ..., t^*+H, across all provinces, where H was the largest horizon considered. To make a prediction for province i at time t^*+h in the mth chain, we draw

$$\hat{y}^m_{t^*+h,i} \sim Poisson(\hat{\lambda}^m_{t^*+h,i} \cdot \hat{y}^m_{t^*+h-1,i})$$

where $\hat{\lambda}^m_{t^*+h,i}$ is computed directly by plugging in the observed and predicted data prior to t^*+h to the fitted model, and we use observed case data in the first step of prediction, i.e. $\hat{y}^m_{t^*,i} = y_{t^*,i}$ for all m. Due to the assumed interrelations between the provinces, we simulated counts for all provinces at a single timepoint before moving on to the next timepoint. For a given prediction horizon h, this process generates an empirical posterior predictive distribution for each province by evaluating the M different predictions for $y_{t^*+h,i}$. Prediction intervals are generated by taking quantiles (e.g., the 2.5% and 97.5%) of this distribution.

1.5 Comparisons of real-time and full-data predictions

As described in the main manuscript, we compared predictions made with available data as if in real-time to predictions made with the final, completely reported dataset. Supplemental Figure B show the real-time and full-data predictions for a selected few provinces.

1.6 Considerations in making real-time, multi-step predictions

Statistical frameworks to create multi-step predictions of time-series data exist [1, 2], but have seen limited use for real-time predictions in public health settings. Creating a statistical model to create multi-step forecasts (i.e. not just predicting the 'next' value in a time-series, but a sequence of future values at different time horizons) raises methodological considerations that are not present when just

Figure B: Comparison between real-time forecasts (red lines and triangles) and full-data forecasts (black lines and circles) for Bangkok. Fully observed case counts are shown as vertical bars. The graph is faceted by analysis date, with each separate plot showing predictions made on a particular analysis date. The first four rows represent predictions whose analysis date was in 2013.

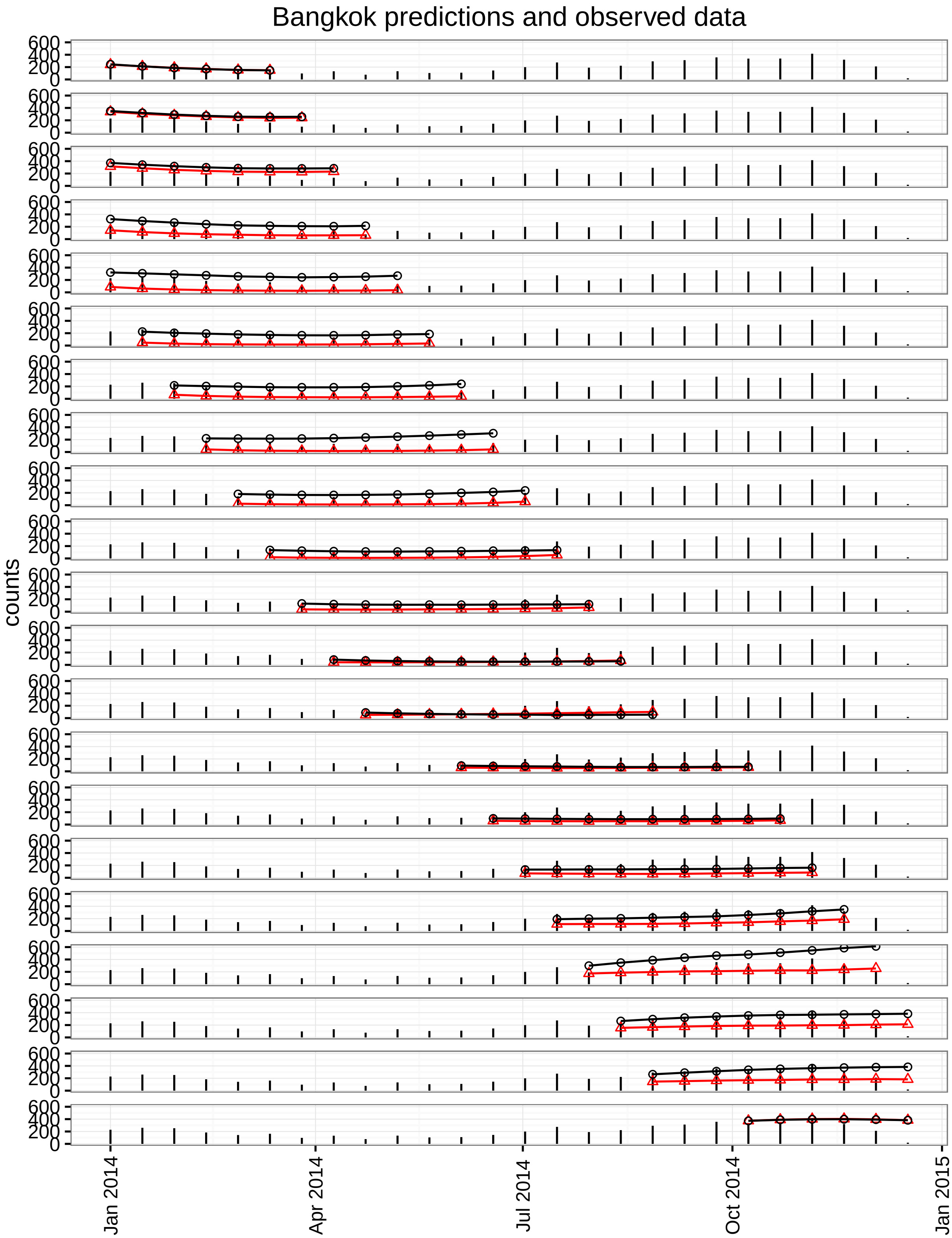

predicting a single time step forward. For example, one may use "recursive" methods to generate a dependent trajectories of the time series or "direct" methods that use a model explicitly predict the entire trajectory as independent observations [2]. Additionally, evaluation becomes more complex, as the performance of the model at each prediction horizon must be evaluated separately. Research on time-series prediction has examined the bias and variance in theoretical settings of different methods for multi-step predictions [3], although little guidance exists on how best to implement multi-step predictions in appled settings. Our current model uses a recursive method for generating predictions.

One critical and unique challenge in real-time forecasting efforts that are used to inform public health decision-making is how to evaluate forecasts when the forecasts themselves are being used to inform decision-making about interventions. For example, if a forecast is made that predicts higher than usual incidence and an effective intervention is put in place that decreases transmission, it would appear that the original forecast was wrong. This scenario represents a substantial public health victory for forecasting: the forecasts were right and they enabled a timely intervention. However, it is difficult to observe the forecasting victory here because it looks as though the forecast of high incidence was incorrect. One way to address this challenge would be to create multi-scenario forecasts that take into account different possible public health responses. This would be a crucial step both for being able to appropriately assess the accuracy of forecasts when interventions are used and to evaluate the effectiveness of interventions. Without including this feature in real-time predictions, a forecast made pre-intervention may end up looking incorrect despite it being an important factor that drove action being taken.

2 Province-level factors that influenced predictive performance

We ran several analyses to identify province-level characteristics that influenced local predictive performance. Factors considered included the following province-specific measures:

- the total cases observed in 2014,
- the ratio between the number of cases reported in 2014 and the median annual cases,
- a measure of seasonality,
- residual variance once seasonality was accounted for,
- the fraction of cases with a reporting delay of greater than 3 months (6 biweeks), and
- the population density.

Unadjusted relationships between the log-scale relative MAE and each predictor of interest are shown in Figure C. Seasonality was determined by fitting a Poisson generalized additive model with a cyclical smooth spline on time-of-year to observed case data. The maximum magnitude of the seasonal effect (standardized across all seasons) is used as a measure of strength of seasonality. Additionally, the standard deviation of the residuals from this model is used as a measure of residual variability. Due to

the limited number of observations relative to the number of predictors of interest, we do not present the results from multivariable models.

Figure C: Relationships between possible factors influencing prediction accuracy on the population level.

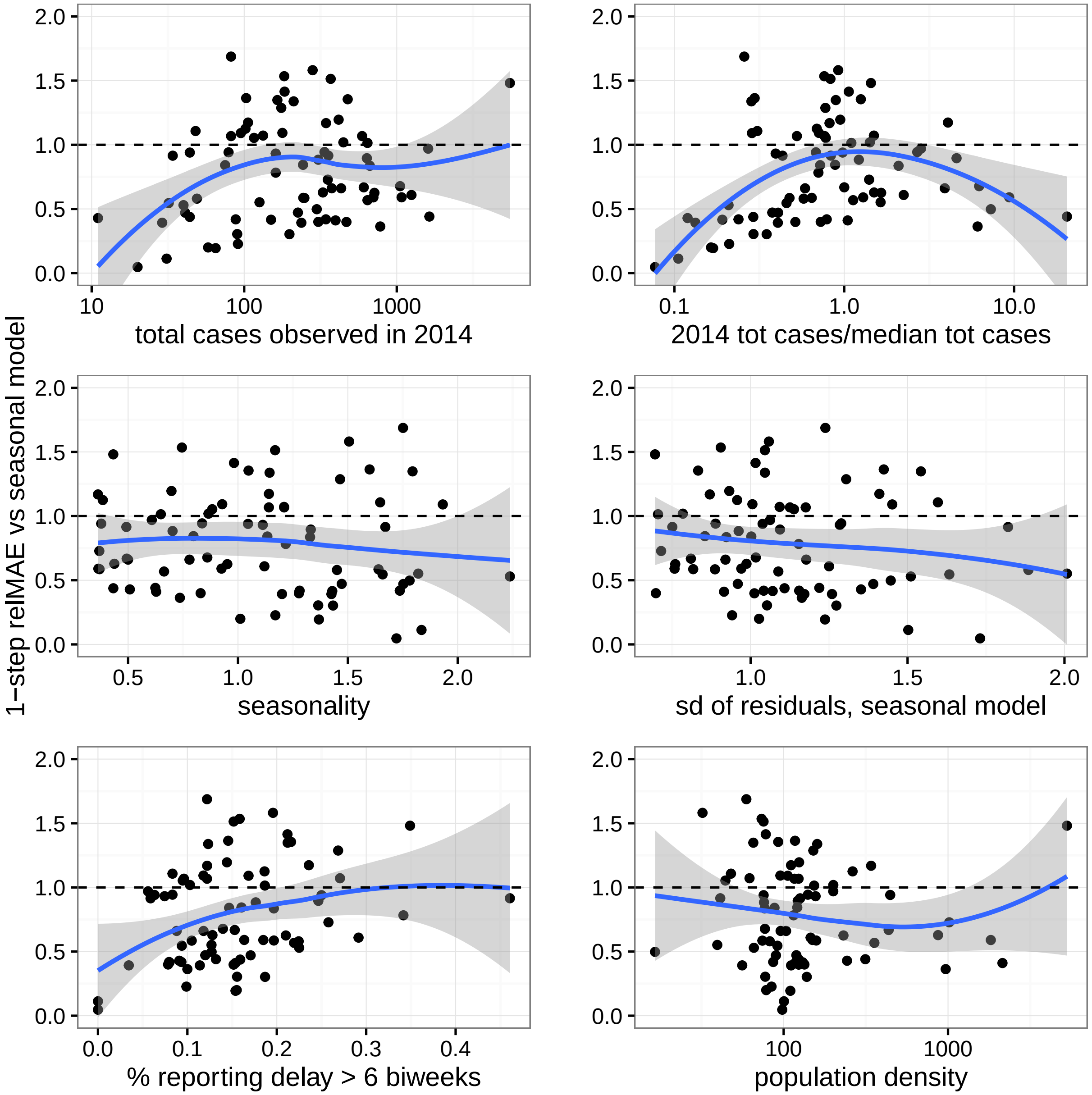

References

[1] Jeffrey Shaman, Alicia Karspeck, Wan Yang, James Tamerius, and Marc Lipsitch. Real-time influenza forecasts during the 2012–2013 season. *Nature Communications*, 4, December 2013.

[2] Souhaib Ben Taieb, Gianluca Bontempi, Amir F Atiya, and Antti Sorjamaa. A review and comparison of strategies for multi-step ahead time series forecasting based on the NN5 forecasting competition. *Expert Systems with Applications*, 39(8):7067–7083, June 2012.

[3] Jianqing Fan and Qiwei Yao. Nonlinear Prediction. In *Nonlinear Time Series*, pages 441–486. Springer Science & Business Media, September 2008.

		cases reported	
delivery date	analysis date	new	cumulative
2014-01-16	2014-01-29	24	0
2014-01-24	2014-01-29	156	1
2014-02-06	2014-02-12	328	2
2014-02-19	2014-02-26	808	4
2014-03-05	2014-03-12	483	6
2014-03-20	2014-03-26	306	7
2014-04-04	2014-04-09	366	8
2014-04-10	2014-04-23	2	8
2014-04-20	2014-04-23	290	9
2014-05-02	2014-05-07	274	10
2014-05-17	2014-05-21	291	11
2014-05-29	2014-06-04	415	13
2014-06-13	2014-06-18	576	15
2014-06-26	2014-07-02	2516	23
2014-07-11	2014-07-16	1314	27
2014-08-15	2014-08-27	2114	35
2014-08-18	2014-08-27	2089	42
2014-09-05	2014-09-10	1003	45
2014-09-18	2014-09-24	917	48
2014-09-30	2014-10-08	4552	64
2014-10-16	2014-10-22	1867	70
2014-10-28	2014-11-05	900	73
2014-11-13	2014-11-19	1091	77
2014-12-17	2015-01-01	920	80
2014-12-19	2015-01-01	3184	90
2014-12-26	2015-01-01	1127	94
2015-01-10		538	96
2015-01-24		436	97
2015-04-30		749	100

Table B: Dates of dengue data deliveries and analyses in 2014. For each delivery, the number of new cases delivered and the cumulative percent of total cases for the year is also shown. Analyses were run on the first day of each biweek only when new data was delivered in the previous biweek.

id	name	pop'n	median annual cases	total 2014 cases	% cases delivered $\geq$ 6 biweeks
TH01	Mae Hong Son	209,153	41	299	13
TH02	Chiang Mai	1,737,041	435	343	9
TH03	Chiang Rai	1,172,928	294	31	0
TH04	Nan	452,814	77	126	13
TH05	Lamphun	412,741	70	32	9
TH06	Lampang	743,143	318	82	12
TH07	Phrae	427,398	185	165	21
TH08	Tak	526,382	305	281	20
TH09	Sukhothai	629,707	251	178	12
TH10	Uttaradit	438,578	218	29	3
TH11	Kamphaeng Phet	797,391	381	477	22
TH12	Phitsanulok	912,827	433	91	10
TH13	Phichit	548,242	275	243	16
TH14	Phetchabun	940,076	521	248	10
TH15	Uthai Thani	297,493	149	116	9
TH16	Nakhon Sawan	992,749	735	432	12
TH17	Nong Khai	458,772	226	175	27
TH18	Loei	546,028	156	48	8
TH20	Sakon Nakhon	941,810	260	20	0
TH22	Khon Kaen	1,741,980	743	211	12
TH23	Kalasin	824,538	369	88	8
TH24	Maha Sarakham	827,639	408	161	7
TH25	Roi Et	1,084,985	783	150	8
TH26	Chaiyaphum	963,907	445	369	15
TH27	Nakhon Ratchasima	2,525,975	1,127	593	10
TH28	Buri Ram	1,274,921	908	468	15
TH29	Surin	1,122,900	567	198	19
TH30	Si Sa Ket	1,055,980	597	225	12
TH31	Narathiwat	670,002	115	1,076	16
TH32	Chai Nat	305,587	151	44	16
TH33	Sing Buri	199,982	92	11	9
TH34	Lop Buri	769,925	439	416	14
TH35	Ang Thong	254,292	148	102	19
TH36	Phra Nakhon Si Ayutthaya	870,671	420	344	12
TH37	Saraburi	717,054	316	447	10
TH38	Nonthaburi	1,334,083	379	397	15
TH39	Pathum Thani	1,327,147	219	328	13
TH40	Bangkok Metropolis	8,305,218	3,843	5,518	35
TH41	Phayao	417,380	192	40	22
TH42	Samut Prakan	1,828,694	544	703	18
TH43	Nakhon Nayok	246,868	107	82	12
TH44	Chachoengsao	715,603	421	306	8
TH46	Chon Buri	1,555,358	570	643	22
TH47	Rayong	821,072	433	714	21
TH48	Chanthaburi	485,611	319	666	20
TH49	Trat	247,876	104	75	15
TH50	Kanchanaburi	801,519	427	356	46
TH51	Suphan Buri	845,561	379	244	20
TH52	Ratchaburi	796,748	584	643	19
TH53	Nakhon Pathom	943,892	608	608	15
TH54	Samut Songkhram	185,564	116	79	6
TH55	Samut Sakhon	887,191	252	353	26
TH56	Phetchaburi	472,589	251	306	18
TH57	Prachuap Khiri Khan	467,466	240	183	16
TH58	Chumphon	467,801	173	184	21
TH59	Ranong	249,017	45	44	25
TH60	Surat Thani	1,009,351	353	58	16
TH61	Phangnga	258,535	89	133	27
TH62	Phuket	525,709	128	780	10
TH63	Krabi	362,203	169	1,052	14
TH64	Nakhon Si Thammarat	1,450,466	558	1,249	29
TH65	Trang	598,877	139	637	25
TH66	Phatthalung	480,976	125	336	8
TH67	Satun	274,863	26	106	24
TH68	Songkhla	1,481,021	568	1,607	6
TH69	Pattani	609,015	80	1,637	13
TH70	Yala	433,167	96	375	9
TH72	Yasothon	487,976	347	103	15
TH73	Nakhon Phanom	583,726	332	95	17
TH74	Prachin Buri	546,996	228	161	34
TH75	Ubon Ratchathani	1,746,790	583	237	11
TH76	Udon Thani	1,288,365	384	65	15
TH77	Amnat Charoen	283,732	100	41	17
TH78	Mukdahan	357,339	85	49	22
TH79	Nong Bua Lam Phu	485,974	78	34	6
TH80	Sa Kaeo	555,961	308	90	16

Table C: Summary data on all 77 provinces of Thailand.

Table D: Split provinces

Province	Type	Family	Founding Year	First Data Year
Chiang Rai	Parent	Chiang Rai	pre-1968	1968
Phayao	Child	Chiang Rai	1977	1978
Nong Khai	Parent	Nong Khai	pre-1968	1969
Bueng Kan	Child	Nong Khai	2011	2011
Prachin Buri	Parent	Prachin Buri	pre-1968	1968
Sa Kaeo	Child	Prachin Buri	1993	1999
Ubon Ratchathani	Parent	Ubon Ratchathani	pre-1968	1968
Yasothon	Child	Ubon Ratchathani	1972	1972
Amnat Charoen	Child	Ubon Ratchathani	1993	1999
Udon Thani	Parent	Udon Thani	pre-1968	1968
Nong Bua Lamphu	Child	Udon Thani	1993	1999
Nakhon Phanom	Parent	Nakhon Phanom	pre-1968	1968
Mukdahan	Child	Udon Thani	1982	1999
